# Effect of Preparation Conditions of Fe@SiO_2_ Catalyst on Its Structure Using High-Pressure Activity Studies in a 3D-Printed SS Microreactor

**DOI:** 10.3390/molecules30020280

**Published:** 2025-01-13

**Authors:** Meric Arslan, Sujoy Bepari, Juvairia Shajahan, Saif Hassan, Debasish Kuila

**Affiliations:** 1 Department of Applied Science and Technology, North Carolina Agricultural and Technical State University, Greensboro, NC 27411, USA; marslan@ncat.edu; 2 Department of Chemistry, North Carolina Agricultural and Technical State University, Greensboro, NC 27411, USA; sbepari@ncat.edu (S.B.); shassan@aggies.ncat.edu (S.H.); 3 Joint School of Nanoscience and Nanoengineering, North Carolina Agricultural and Technical State University, Greensboro, NC 27411, USA; jshajahan@aggies.ncat.edu

**Keywords:** FTS, SS microreactor, core–shell Fe catalyst, mesoporous composite oxide

## Abstract

Fischer–Tropsch synthesis (FTS) in a 3D-printed stainless steel (SS) microchannel microreactor was investigated using Fe@SiO_2_ catalysts. The catalysts were prepared by two different techniques: one pot (OP) and autoclave (AC). The mesoporous structure of the two catalysts, Fe@SiO_2_ (OP) and Fe@SiO_2_ (AC), ensured a large contact area between the reactants and the catalyst. They were characterized by N_2_ physisorption, H_2_ temperature-programmed reduction (H_2_-TPR), scanning electron microscopy (SEM), transmission electron microscopy (TEM), X-ray diffraction (XRD), Fourier-transform infrared spectroscopy (FTIR), X-ray photoelectron microscopy (XPS), and thermogravimetric analysis–differential scanning calorimetry (TGA-DSC) techniques. The AC catalyst had a clear core–shell structure and showed a much greater surface area than that prepared by the OP method. The activities of the catalysts in terms of FTS were studied in the 200–350 °C temperature range at 20-bar pressure with a H_2_/CO molar ratio of 2:1. The Fe@SiO_2_ (AC) catalyst showed higher selectivity and higher CO conversion to olefins than Fe@SiO_2_ (OP). Stability studies of both catalysts were carried out for 30 h at 320 °C at 20 bar with a feed gas molar ratio of 2:1. The Fe@SiO_2_ (AC) catalyst showed higher stability and yielded consistent CO conversion compared to the Fe@SiO_2_ (OP) catalyst.

## 1. Introduction

FTS entails the catalytic conversion of synthesis gas (syngas (CO+H_2_)) into long-chain hydrocarbons. This synthesis was named in 1923 after Franz Fischer and Hans Tropsch, who discovered the synthesis of hydrocarbons over Co catalysts [1]. Syngas can be obtained from different carbon-based materials (natural gas, biogas, biomass, coal, and waste) by different reforming processes [2,3,4,5,6,7]. Biomass can be converted into synthesis gas by single-step and two-step processes [8,9,10,11]. The FTS process is necessary because of the production of liquid hydrocarbon fuels, like diesel and gasoline from syngas. The fuel produced by the FTS process is of high quality due to an absence of sulfur and nitrogenous compounds. A high cetane number of the diesel fraction is produced, which results superior combustion properties and emissions. Carbon recycling potential and production of clean fuels are the advantages of this process. The disadvantages of this process are high capital and operating costs, low overall efficiency, environmental concerns, catalyst sensitivity, and hydrogen demand. The FTS process is initiated by the dissociation of CO and H_2_ on the catalyst surface, followed by hydrogenation and polymerization [12,13,14]. However, the exact stepwise mechanism of FTS remains unknown [15]. The product selectivity of the process is explained by the Anderson–Schulz–Flory (ASF) model [16], and the product’s molecular weight distribution is governed by the probability factor α. A high value of α (>0.90) is considered to produce the desired product with maximum selectivity for heavy hydrocarbons, which can be easily converted to liquid fuels. Catalysts are required for FTS to control the chemical reactions for conversion of syngas into hydrocarbons. The role of the catalyst is very important because of (i) activation of syngas; (ii) selective hydrocarbon formation; (iii) control of reaction pathways; (iv) enhanced reaction rate; (v) optimization of process parameters (temperature and pressure); (vi) product distribution; and (vii) increased carbon efficiency. Thus, catalysts play an important role in demonstrating the economic and environmental feasibility of the FTS process.

The three most commonly used active metals in FTS are Co, Fe, and Ru. Fe-based catalysts have distinct advantages: (i) they are inexpensive, highly suitable for industry, and broadly available; (ii) they facilitate water–gas shift reactions; and (iii) they have a comparatively elevated activation temperature such that their product selectivity is favorable towards short-chain oxygenates, gasoline fractions, and short-chain unsaturated hydrocarbons [17,18]. To improve Fe-based catalysts’ activity and enhance mechanical stability, a mesoporous support was used [19,20]. However, the formation of mixed oxides is the main disadvantage of these supports, which are very difficult to reduce, thus decreasing their catalytic activity. However, it is difficult to obtain high loading of active metals over the supports, which also inhibits the catalyst activity for FTS processes [18]. In the case of silica-supported Fe-based catalysts, iron silicate cannot be reduced completely to form α-Fe as observed by Wielers et al. [21]. Zhang et al. [22] also studied the design of iron silicate, which decreased the reduction of iron oxide and showed lower activity in FTS. This Fe–SiO_2_ interaction becomes very critical in the formation of α-Fe catalyst to optimize its stability and activity [23].

Core–shell catalysts provide a robust alternative for enhancing catalyst stability and activity. In recent years, core–shell catalysts have gained more awareness due to the “severe confinement effect” of encasing the active metal core in a shell, as well as the unique pore structure and acidic properties of the shell itself, which can increase the desired product selectivity and also protect the catalyst core during the reactions [24]. Core–shell catalysts have advantages such as: (i) intensifying or providing new chemical or physical capabilities; (ii) limiting volume extension and maintaining formation integrity; (iii) protecting the core from summation into large particles; and (iv) percolating ions or molecules onto the core, which improves selectivity [25]. It has been shown that consolidating nanoscale catalysts need to be converted into oxide shells in order to significantly increase their thermal stability during reactions. Song et al. [26] prepared differently structured FeMn@HZSM-5 catalysts for FTS to olefin (FTO) reactions [27]. The core–shell-structured catalyst with HZSM-5 shell and FeMn core showed pretty good catalytic activity in terms of higher olefin and lower CO_2_ selectivity. They observed that the HZSM-5 in the shell part could hinder H_2_O diffusion and reduce the rate of the water–gas shift (WGS) reaction in the FTO reaction. Ni et al. [28] investigated potassium (K)- and graphite carbon (GC)-promoted Fe-based catalyst for the FTS reaction. They observed that GC and K improved the catalyst’s activity. The GC was used as a reducing agent for synthesizing metallic Fe^0^ from Fe^3+^, and effectively increased increases the strength of SiO_2_ channels. However, the addition of potassium increased the CO chemisorption and inhibited H_2_ chemisorption, which resulted in lower hydrogenation capacity and increased olefin selectivity. Ni et al. modified a Fe@SiO_2_ catalyst with GC and used it for FTS-to-olefins (FTO) studies [23]. The addition of GC affected the Fe–SiO_2_ interaction. The rigid porous structure of GC facilitated access to synthesis gas and prevented the collapse of the mesoporous channel during the FTO response time. Qiu et al. [29] synthesized a SAPO-34 zeolite shell with a Fe_3_C core catalyst for FTS.

For mesoporous shell structures, the main goal is engineering that allows reactants to diffuse to the core surface so that the catalytically energetic phase can be produced. Given their potential to enhance both stability and activity, core–shell-structured catalysts were selected for an in-depth analysis of their chemical and physical properties on FTS reactions. In this study, iron-based catalysts were synthesized via one-pot (OP) hydrothermal and autoclave (AC) methods. While the Fe@SiO_2_ (AC) catalyst was synthesized at high pressure and 200 °C, the Fe@SiO_2_ (OP) catalyst was prepared at room temperature under atmospheric pressure. The catalysts were characterized using BET, SEM-EDS, TEM, XRD, XPS, FTIR, TPR, and TGA-DSC techniques. The comparative evaluation of their catalytic performance was conducted in FTS reactions using 3D-printed SSMR at 20 bar.

## 2. Results and Discussion

### 2.1. BET Analysis

**Figure 1:**
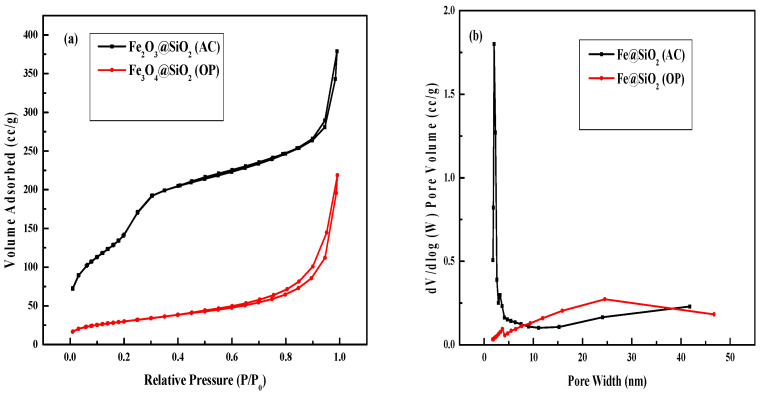
N_2_ Adsorption–desorption isotherms (**a**) and pore size distribution plots (**b**) of Fe@SiO_2_ (AC) and Fe@SiO_2_ (OP) catalysts.

Nitrogen BET physical adsorption analysis was performed to examine the surface properties of the catalyst. In Figure 1a, the BET isotherm for the Fe@SiO_2_ (AC) catalyst exhibits a typical type IV isotherm with a large hysteresis loop, indicative of mesoporous structures as classified by the International Union of Pure and Applied Chemistry (IUPAC). The absence of capillary condensation in the Fe@SiO_2_ (AC) catalyst indicates that the synthesis method and process have a significant effect on the resulting structural properties of the catalyst.

In the case of the Fe@SiO_2_ (OP) catalyst, an H_3_ hysteresis loop with capillary condensation is observed, indicating a mixture of typical microporous and mesoporous structures [30,31]. Desorption data were used for pore size distribution plots. The pore size distribution of Fe@SiO_2_ (AC) catalyst mainly indicates pores of approximately 2.09 nm, confirming that the catalyst has a mesoporous structure. In Figure 1b, lower BJH pore size distribution observed for the Fe@SiO_2_ (OP) catalyst indicates it to be a nonporous material, and it also has lower N_2_ adsorption capacity [30].

The catalyst surface area, average pore diameter, and pore volume of the catalysts are presented in Table 1. The Fe@SiO_2_ (OP) catalyst exhibits the lowest surface area, attributed to its larger pore diameter of 12.6 nm relative to other catalysts. Conversely, the Fe@SiO_2_ (AC) catalyst displays the highest surface area and the smallest pore diameter among the catalysts studied. As such, the catalyst synthesis procedure plays an important role in controlling the physical morphology of the catalyst. The higher temperature used in the autoclave method might be the main factor in generating the lower pore diameter of the catalyst.

### 2.2. XRD Analysis

**Figure 2:**
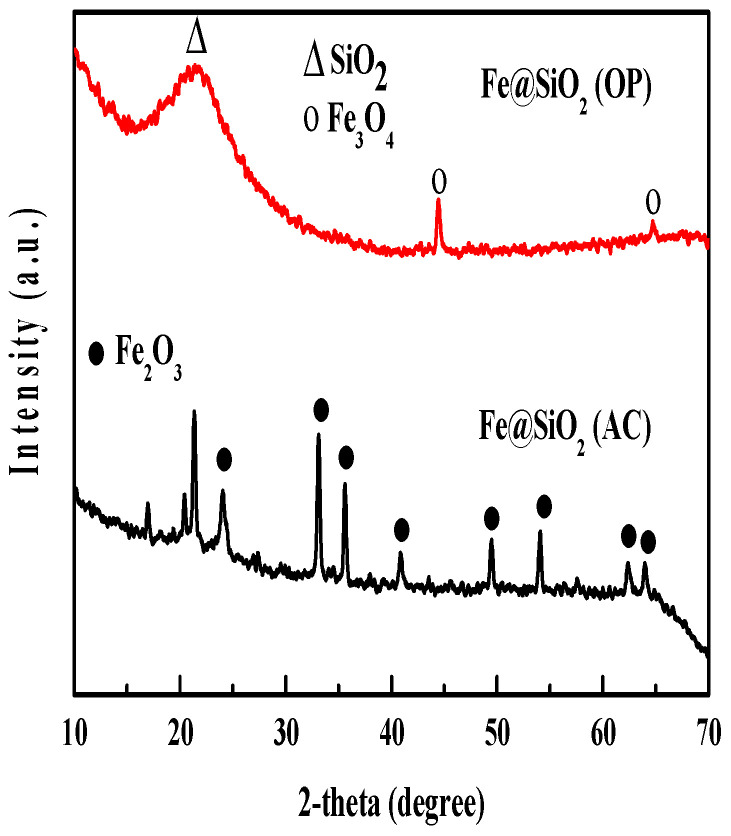
Wide-angle XRD patterns of Fe@SiO_2_ (AC) and Fe@SiO_2_ (OP) catalysts.

The XRD patterns of all catalyst samples are shown in Figure 2.

The composition and crystal structure of the catalysts were analyzed from XRD studies. As presented in Figure 2, the Fe_2_O_3_ diffraction peaks with rhombohedral structure are observed at 23.97° (012), 33.15° (104), 35.6° (110), 40.9° (113), 49.45° (024), 54.19° (116), 62.36° (214) and 63.94° (300) for the Fe@SiO_2_ (AC) catalyst. The observed diffraction peaks match with those observed for the Fe_2_O_3_ structure (JCPDS-24-0072) [32]. For the Fe@SiO_2_ (OP) catalyst, an orthorhombic Fe_3_O_4_ structure with characteristic peaks observed at 44.57° (131) and 64.9° (144), as indexed in the JCPDS-89-6466 database, is found. The broad peak around 22° corresponds to mesoporous SiO_2_ and is consistent with previous studies reported in the literature [33,34,35]. The Fe@SiO_2_ (OP) catalyst shows only two peaks of Fe_3_O_4_. This suggests that the catalyst is almost amorphous in nature.

The average crystal grain size for each catalyst was calculated from XRD data using the modified Scherrer equation, as detailed in [36], and are presented in Table 2. Specifically, the average crystal size of Fe_3_O_4_ in the Fe@SiO_2_ (OP) catalyst is 20.34 nm, while the average crystal size of Fe_2_O_3_ in the Fe@SiO_2_ (AC) catalyst is 20.45 nm.

### 2.3. SEM Analysis

**Figure 3:**
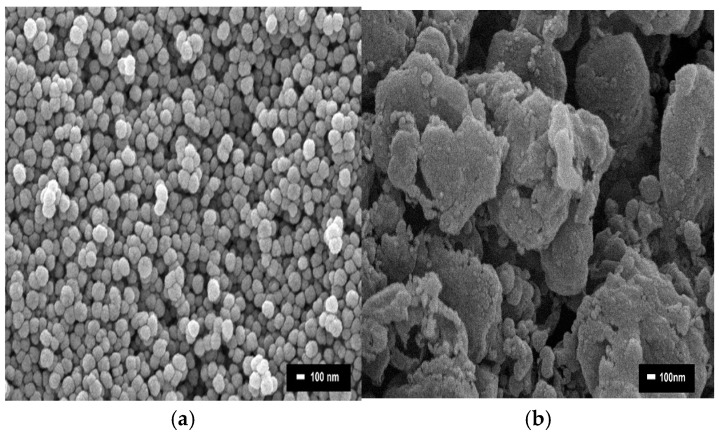
SEM images of catalysts: (**a**) Fe@SiO_2_ (AC); (**b**) Fe@SiO_2_ (OP).

The core–shell catalysts’ morphologies and elemental composition (wt. %) were obtained using SEM. Figure 3 exhibits the SEM images of all core–shell catalysts. Figure 3a shows the shape of the particles to be spherical in Fe@SiO_2_ (AC) catalyst [37]. In Figure 3b, the particles become larger due to agglomeration and tend to form irregularly shaped spheres produced by the one-pot method (Fe@SiO_2_ (OP)) [38]. The particles are well distributed throughout the support.

Table 3 presents the EDS analysis of all catalysts. The EDS results show that the Fe content of both catalysts (Fe@SiO_2_ (OP) and Fe@SiO_2_ (AC)) is almost the same. The highest Si content is observed in Fe@SiO_2_ (AC). This indicates that the method of preparation could influence fast infiltration of Si in the shell part of the catalyst [39].

### 2.4. TEM Analysis

**Figure 4:**
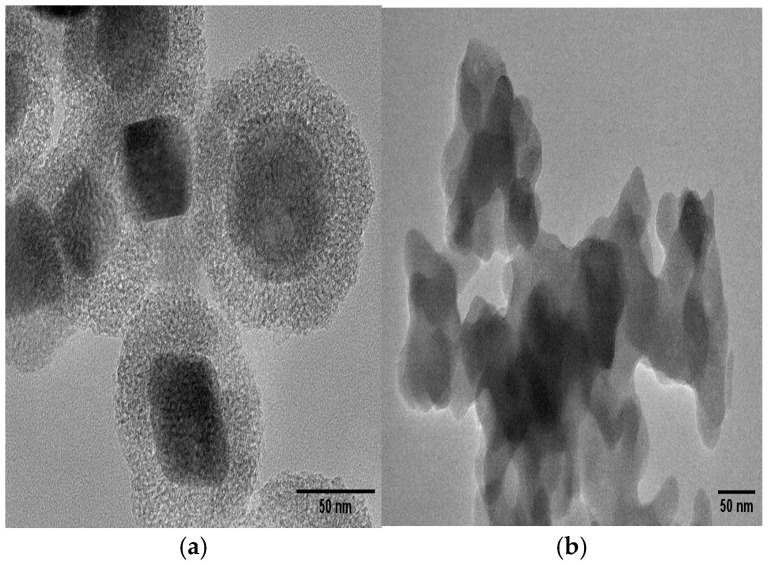
TEM images of catalysts: (**a**) Fe@SiO_2_ (AC); (**b**) Fe@SiO_2_ (OP).

The TEM images of the core–shell nanoparticles are presented in Figure 4, with particle diameters quantified using ImageJ software (Version 1.54). Figure 4a displays the TEM image of the Fe@SiO_2_ (AC) catalyst, revealing a core diameter of 22.13 nm for Fe_2_O_3_ and a shell thickness of 15.12 nm. This TEM analysis supports the XRD data presented before. Figure 4b shows the Fe@SiO_2_ (OP) catalyst that according to XRD analysis exhibits an almost amorphous structure. As such, it is very difficult to measure the particle diameter of this sample by TEM analysis. Agglomeration of catalyst particles is observed in the transmission electron microscopy images and is consistent with the SEM studies. The size difference depending on the type of metal is due to the features of SiO_2_ [39]. This is verified by EDS analysis. The Fe@SiO_2_ (AC) and Fe@SiO_2_ (OP) catalysts show differences between iron and silicon. More specifically, the Fe@SiO_2_ (AC) catalyst clearly shows distribution of iron and silica compared to the iron-based catalysts. In summary, the autoclave method is significantly better than the one-pot method for the synthesis of core–shell catalysts.

### 2.5. TPR Analysis

**Figure 5:**
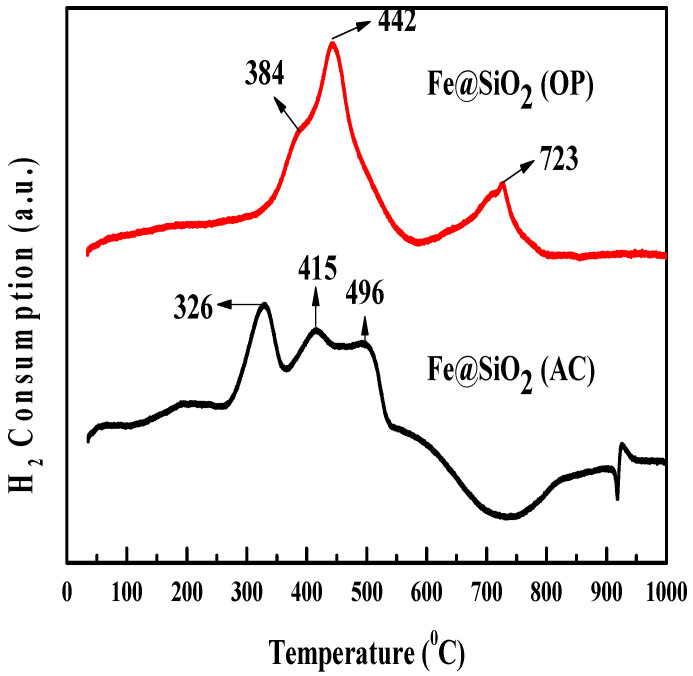
H_2_-TPR analyses of Fe@SiO_2_ catalysts prepared by one-pot and autoclave procedures.

Temperature Programmed Reduction (TPR) studies using H_2_ were performed to consider the reduction of the metal–support and metal oxide interactions. Figure 5 shows the H_2_-TPR profiles. The temperature-programmed reduction profile of Fe@SiO_2_ (AC) catalyst shows hydrogen consumption peaks observed at 326 °C and 496 °C, attributed to the two sequential reductions of Fe_2_O_3_ to Fe_3_O_4_ (Fe_2_O_3_ → Fe_3_O_4_) and Fe_3_O_4_ to Fe (Fe_3_O_4_ → FeO + Fe), respectively [23,40,41,42]. The second peak (415 °C) corresponds to strong interaction between Si-OH groups with the FeO of the mesoporous SiO_2_ support [23]. For the Fe@SiO_2_ (OP) catalyst, the reduction peak of 350–800 °C indicates reduction of magnetite to metallic iron via iron oxide in the presence of the supporting structure [43,44,45,46]. In Figure 5, large and broad peaks at 442 °C and 723 °C with a small shoulder peak at 384 °C are observed. This is different from that of pure hematite (Fe_2_O_3_) [43,44,46], but similar to that observed in Fe/SiO_2_ catalysts [47,48]. This indicates that the presence of Si in the catalysts played an important role as a supporting structure in the TPR process and correlates with the BET analysis (Table 1).

Table 4 highlights the degree of reduction and hydrogen consumption for various core–shell catalysts. Notably, the Fe@SiO_2_ (OP) catalyst exhibited the highest hydrogen consumption, indicating a higher concentration of metal oxide species that can be reduced. The reduction was assessed within the range 150–400 °C, relevant to the FTS reactions [49]. The Fe@SiO_2_ (AC) catalyst achieved the highest reduction degree, at 55.64%, indicating the most substantial reduction in iron oxide species within this temperature range. Conversely, the Fe@SiO_2_ (OP) catalyst demonstrated a lower reduction degree of 14.11%, underscoring the influence of the synthesis method on the reduction of metal oxides in core–shell catalysts.

### 2.6. TGA-DSC Analysis

**Figure 6:**
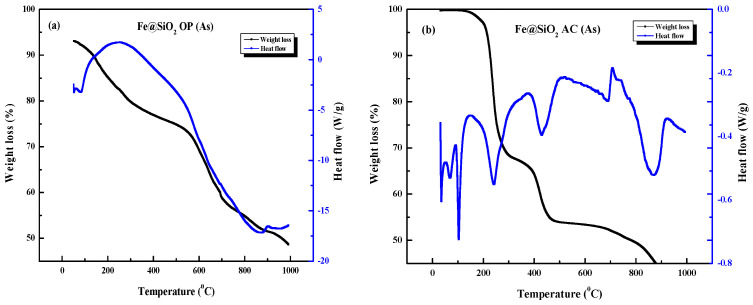
TGA-DSC thermograms of (**a**) Fe@SiO_2_ OP (As); (**b**) Fe@SiO_2_ AC (As) (As = prepared as such).

Figure 6a shows the TGA-DSC analyses of uncalcined Fe@SiO_2_ OP. There are three discrete weight loss slopes, each corresponding to a phase change or glass transition on the heat flow curve. It starts from 25 °C to about 400 °C, the weight loss curve is linear with a negative slope, and the weight loss can be due to decomposition of templating agents from the sample [50]. This corresponds to a local maximum on the heat flow curve, and from 400 °C to 500 °C, the weight loss curve is relatively flat, with a slight downwards slope. The weight loss in the sample is from the loss of templating agents and due to crystal formation. As such, the weight loss is relatively stable due to crystallization, evidenced by the local maximum on the heat flow curve at 250 °C of the Fe@SiO_2_ (OP) catalyst [51]. From 600 °C to 1000 °C, there is rapid weight loss corresponding to an endothermic peak on the heat flow curve at 900 °C. The weight loss curve is slightly less steep after 700 °C. The weight loss can be based on both templating agents being released from the sample as well as decomposition of the sample, and the less steep portion of the curve can be based on a low rate of evaporation of the templating agent, as most of it would have been removed from the sample by that time [50,52]. The ramp rate of this process was 10 °C/min for a total of approximately 100 min for the TGA-DSC analysis.

The TGA-DSC thermogram of the uncalcined Fe@SiO_2_ (AC), as shown in Figure 6b, revealed a steep, negative slope in the weight curve between 150 °C and 200 °C, comparable to the decomposition of the templating agent [50,52]. The removal of templating agents as well as the thermal decomposition of the sample are responsible for the total weight loss [50,52]. The weight of the sample remains relatively unchanged until the temperature reaches 400 °C, when there is another decrease in weight until about 450 °C. From 450 °C to 800 °C, there is a steady decrease in weight, and the sample weight reduces rapidly from 800 °C to 900 °C. This corresponds to a steep endothermic heat flow from 700 °C, which reaches a local minimum around 900 °C. There are sharp downward peaks at each temperature, where catalyst weight reduces rapidly.

### 2.7. FTIR Analysis

**Figure 7:**
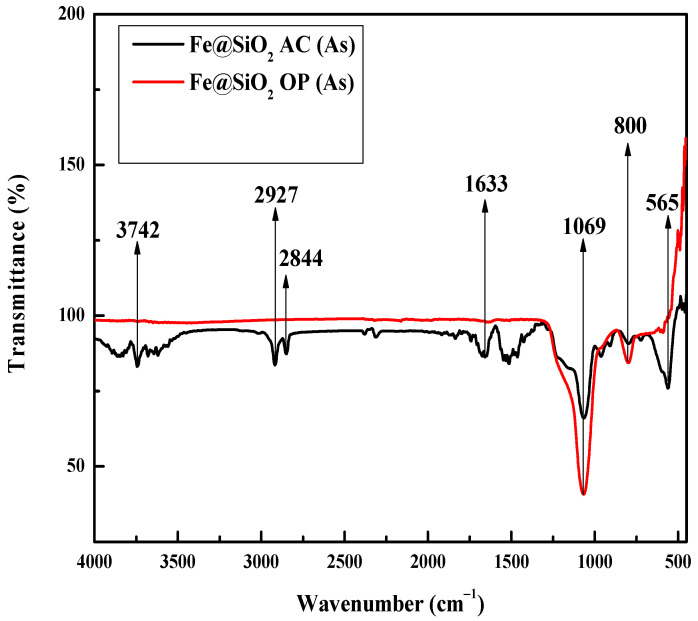
FTIR analysis of all catalysts prior to calcination: Fe@SiO_2_ OP (As); Fe@SiO_2_ AC (As) catalysts (As = as synthesized).

The FTIR analysis of as-synthesized (As) samples is shown in Figure 7. The peak at 565 cm^−1^ in the spectrum is due to Fe-O stretching vibration [53].

The FTIR spectra confirmed the presence of a silica layer on the iron nanoparticles due to the presence of peaks at ~069 and 800 cm^−1^ [54]. In the absence of iron, these peaks are observed at 1100 and 808 cm^−1^. This shift suggests that the strength of the siloxane bridge, Si-O-Si, decreased due to interaction of hydroxyl groups with the iron nanoparticles. This further suggests formation of Si-O-Fe bridges in the Fe@SiO_2_ (OP) (As) and Fe@SiO_2_ (AC) (As) catalysts [55]. The infrared spectral band at 1633 cm^−1^ is associated with the hydroxyl deformation mode of interlayer water molecules [30,56], while bands at 2844 and 2927 cm^−1^ are attributed to the asymmetric and symmetric C-H stretching vibrations [57]. The band in the region of 3000 to 3750 cm^−1^ (here 3742 cm^−1^) is due to the stretching mode of the O-H groups from silanol on the surface of silica or the water adsorbed [55]. In the case of Fe@SiO_2_ (OP) catalysts, the only visible bands are observed at 1069 and 800 cm^−1^. Other bands completely disappeared, presumably due to the synthesis protocols of the OP method. The IR spectra of all calcined catalysts are shown in the Supplementary Section of the manuscript (Figure S1 (See Supplementary File)).

### 2.8. XPS Analysis

XPS analysis of the catalysts provides information on bonding as well as the oxidation states of metal. Deconvolution of C 1s scans of the XPS spectra were used to correct the shortcoming of all elemental spectra with respect to the C-C binding energy of 284.8 eV for adventitious carbon present in Fe@SiO_2_ (AC) and Fe@SiO_2_ (OP).

**Figure 8:**
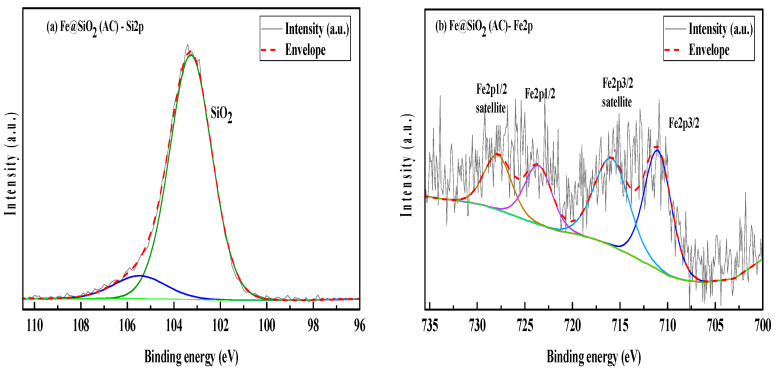
XPS scans of Fe@SiO_2_ (AC): (**a**) Si 2p region (**b**) Fe 2p region.

The C 1s spectrum of Fe@SiO_2_ (AC) showed adventitious carbon in the samples bonded to oxygen (Figure S2 (See Supplementary File)). The C 1s spectrum exhibits peak splitting, which confirms the C-C and C-O-C bonds at 284.8 eV and 286.88 eV, respectively. The oxygen spectrum (Figure S3 (See Supplementary File)), O 1s, has multiple deconvoluted peaks. The peak at a binding energy of 532.94 eV indicates the presence of SiO_2_. The binding energy peak at 532.11 eV confirms the presence of organic C-O. The binding energy peak at 529.40 eV, which represents metal oxide formation [58]. The Si 2p spectrum of Fe@SiO_2_ (AC) (Figure 8a) has peak binding energy of 103.27 eV, showing the presence of SiO_2_, as evidenced by the O 1s spectrum. The peak splitting in SiO_2_ can be ignored due to silicon compound formation. The Fe 2p spectrum (Figure 8b) shows complex multiple splitting, and significant satellite features indicate the presence of mixed oxide. High energy change in binding energy is due to the formation of Fe oxides. The peaks of Fe 2p3/2 and Fe 2p1/2 are observed at the binding energies 711.03 eV and 723.58 eV, respectively. Both peaks have their significant satellite peaks, which are due to oxide formation [59].

The resolution of Si 2p in Fe@SiO_2_ (OP) is lower than that of Fe@SiO_2_ (AC) due to the amorphous nature of Fe@SiO_2_ (OP). The crystalline nature of the Fe@SiO_2_ (AC) has been verified by XRD, as discussed before.

**Figure 9:**
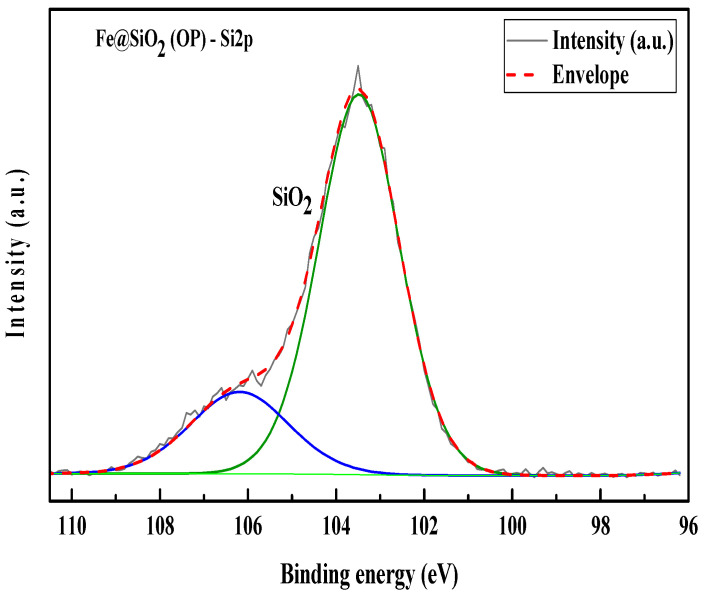
XPS scan of Fe@SiO_2_ (OP): Si 2p region.

The C 1s spectrum of Fe@SiO_2_ (OP) indicates that the adventitious carbon in the samples is bonded to oxygen (Figure S4 (See Supplementary File)). The C 1s spectrum exhibits peak splitting, which confirms C-C and C-O bond presence at 284.79 eV and 287.47 eV, respectively. The oxygen spectrum (Figure S5 (See Supplementary File)), O 1s, has multiple deconvoluted peaks. The peak at binding energy of 533.02 eV indicates the presence of SiO_2_. The binding energy peak at 532.38 eV confirms the presence of organic C-O. The Si 2p spectrum (Figure 9) of Fe@SiO_2_ (OP) has low resolution due to its lower crystallinity. Si 2p has a peak of binding energy at 103.48 eV that indicates the presence of SiO_2_, as confirmed by the O 1s spectrum. The Si 2p spectrum exhibits splitting in Si compounds. Since the SiO_2_ formation is confirmed by the presence of a peak at 103.48 eV, the peak splitting can be ignored.

### 2.9. Fe-Based Core–Shell Catalysts FTS

The FTS reactions were conducted in a 3D-printed SSMR, varying the reaction temperature between 200 °C and 350 °C while maintaining a stable H_2_–CO molar ratio of 2:1 and a GHSV of 12,000 h^−1^ at 20 bar. The N_2_ flow rate was kept at 1.5 mL/min. Hydrocarbon selectivity and CO conversion were evaluated using the equations shown below [56,60] However, CO_2_ formation is important due to WGS reactions. However, it was not considered for hydrocarbon selectivity calculation. CO conversion is affected by the reaction temperature [61].

**Figure 10:**
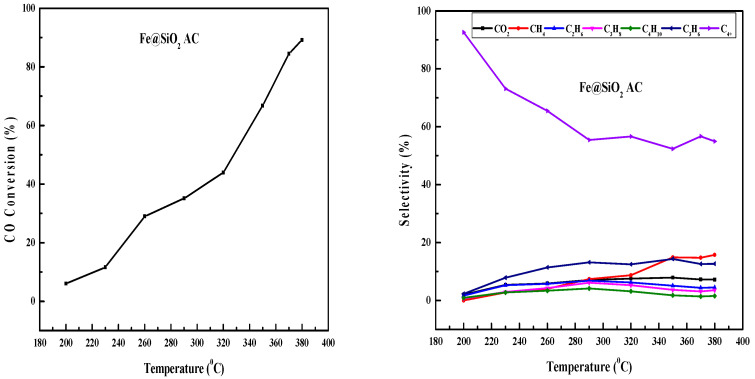
Effect of temperature on CO conversion and product selectivity in FTS using Fe@SiO_2_ (AC) catalyst (conditions: H_2_/CO = 2, 20 bar, GHSV = 12,000 h^−1^, and N_2_ = 1.5 mL/min).

Figure 10 depicts the effect of reaction temperature on CO conversion and product selectivity for the Fe@SiO_2_ (AC) catalyst. A noticeable increase in CO conversion was observed up to 370 °C, suggesting an equilibrium point. Methane selectivity also increased with temperature, with light olefins such as propene showing enhanced selectivity. Conversely, selectivity for ethane, propane, and butane increased with temperature up to 290 °C, but declined at higher temperatures [62]. C_4+_ selectivity decreased up to 290 °C, then almost remained constant at higher temperature. CO_2_ selectivity remained almost unchanged at higher temperatures.

**Figure 11:**
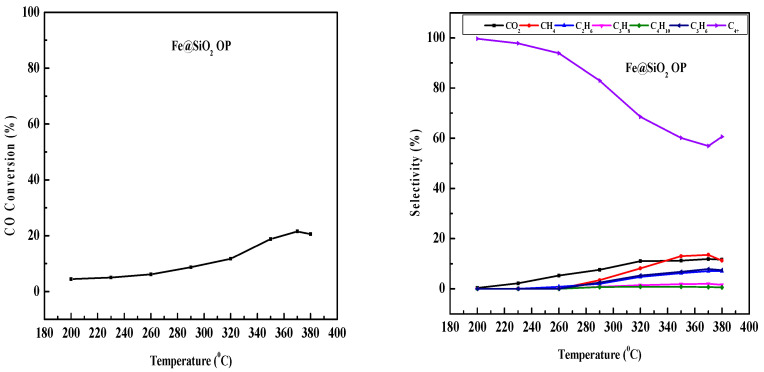
Effect of temperature on CO conversion and product selectivity in FTS using Fe@SiO_2_ (OP) catalyst (conditions: H_2_/CO = 2, 20 bar, GHSV = 12,000 h^−1^, and N_2_ = 1.5 mL/min).

Figure 11 presents the temperature-dependent product selectivity and CO conversion for the Fe@SiO_2_ (OP) core–shell catalyst. CO conversion increased up to 350 °C. After that, it remained unchanged at higher temperature. This reflects that an equilibrium was achieved. As temperature increases, methane and CO_2_ selectivity increases. The constant increase in CO_2_ selectivity across the temperature range indicated active water–gas shift reactions. Moreover, higher yields of light olefins and ethane compared to propane and butane were observed. C_4+_ selectivity decreased with increased reaction temperature.

## 3. Experimental

### 3.1. Materials

The reagents used for catalyst synthesis were of analytical grade with no further purification. Ammonium hydroxide, ACS (American Chemical Society) reagents, and 99% Tetraethyl orthosilicate (TEOS) were obtained from Acros Organics, Fair Lawn, NJ, USA. Co(NO_3_)_2_·6H_2_O, Fe (NO_3_)_3_·9H_2_O, and cetyltrimethylammonium bromide (CTAB) were procured from Sigma Aldrich, St. Louis & Burlington, MA, USA. Acetone and ethanol of ACS grade were purchased from Fisher Scientific, Fair Lawn, NJ, USA.

### 3.2. Fe@SiO_2_ Core–Shell Preparation Using Autoclave (AC) Procedure

The catalysts were prepared in an autoclave with TEOS as the limiting reagent. Quantities of 1.01 g of Fe(NO_3_)_3_, 1.01 g of polyvinylpyrrolidone (PVP), and 2 mL of acetic acid (CH_3_COOH) mixed in 100 mL ethanol at room temperature using magnetic stirring. After that, the solution was moved to the autoclave. The autoclave was controlled at 200 °C for 24 h. Then, the autoclave was allowed to cool to room temperature and the iron oxide nanocubes were obtained.

The as-prepared 100 mL iron oxides in ethanol were mixed in 100 mL deionized water, and an excess 100 mL of ethanol was introduced. Then, 9 mL aqueous ammonia (NH_3 (aq)_) was mixed with this solution to maintain the pH at 9. After that, 0.20 g CTAB was mixed. Finally, 0.625 mL TEOS was added slowly. The solution was stirred at ambient temperature for 24 h, after which the nanoparticles were separated via filtration. The samples were then rigorously first washed with water then with ethanol, followed by drying in an oven at 100 °C. The dried nanoparticles underwent calcination at 500 °C for 6 h in atmospheric conditions to yield the final product [63].

### 3.3. Fe@SiO_2_ Core–Shell Preparation with One-Pot (OP) Synthesis Technique

#### 3.3.1. Synthesis of Fe (Core) Nanoparticles

Scheme S1 (See Supplementary File) demonstrates the preparation of Fe nanoparticles. The procedure is discussed below.

Ferrous chloride and ferric chloride were mixed with sodium hydroxide at room temperature. This procedure yielded iron nanoparticles, as described in the literature [64].

#### 3.3.2. Preparation of SiO_2_ (Shell)

Scheme S2 (See Supplementary File) demonstrates the SiO_2_ shell preparation. The procedure is discussed below.

Oleic acid and chloroform were added to distilled (DI) water. Then, cetyltrimethylammonium bromide (CTAB) was added and heated to 65 °C. Sodium hydroxide (NaOH) was mixed to this solution to maintain the pH. Finally, tetraethoxysilane (TEOS) and ethyl acetate (EtOAc) were added slowly and heated to 70 °C. After 2 h, Fe and SiO_2_ core–shell particles were centrifuged, washed with ethanol, and then dried at 100 °C in an oven [65].

### 3.4. 3D-Printed Stainless Steel Microchannel Microreactor (SSMR)

**Figure 12:**
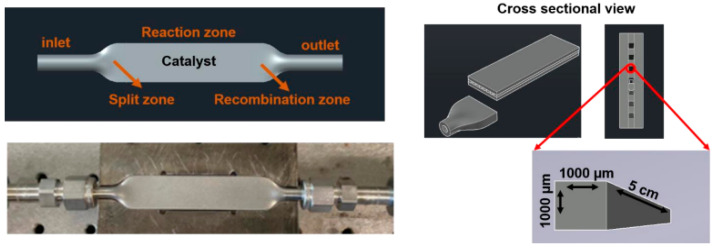
3D-printed stainless steel microchannel microreactor. (Red arrow signify the dimension of each microchannel.)

For high-pressure FTS, the microreactor used to analyze the catalytic reaction was adjusted at the inlet and outlet to provide proper closing. Figure 12 shows the design of the AutoCAD and the final version of the 3D-printed SS microreactor. As shown below, the SS microreactor has seven microchannels between the cylindrical inlet and outlet. Each microchannel is 1000 µm wide, 1000 µm deep, and 5 cm long. The Swagelok tubing aligned perfectly with a Swagelok filter to provide a leak-proof sealing for high-pressure reactions with use of a ¼-inch outer diameter inlet and outlet of the microchannel microreactor [65].

### 3.5. Characterization Techniques

The surface properties of all catalysts were analyzed using N_2_ adsorption-desorption isotherms obtained with a BET surface area analyzer (Model: 3Flex, Make: Micromeritics, Norcross, GA, USA). The experiment was performed at a constant temperature corresponding to liquid N_2_ (−196 °C). Surface area and pore size distributions were calculated from these isotherms using the Barrett–Joyner–Halenda (BJH) and Brunauer–Emmett–Teller (BET) methods. Crystallinity and composition were assessed through X-ray diffraction using a powder X-ray diffractometer (Model: Bruker AXS, Billerica, MA, USA), with a detection range of 10–70° at 0.02° step intervals using Cu Kα1 radiation (wavelength: 1.5406 Å). Temperature-programmed reduction (TPR) experiments using H_2_ helped to determine the oxidation states and reducibility of the catalyst metals. TPR was carried out with a 10% H_2_/Ar (1:9 *v*/*v*) flow at 50 mL/min and a temperature range of room temperature to 800 °C.

Morphological and topographical analyses of the synthesized materials were conducted using a Zeiss Auriga focused ion beam scanning electron microscope (FIB SEM) at the Joint School of Nanoscience and Nanoengineering. Further study of structural properties was conducted using a transmission electron microscope (TEM, Thermo Fisher Talos Model: F200X, Fair Lawn, NJ, USA) operated at 200 kV.

Chemical states and bonding energies were quantified via X-ray photoelectron spectroscopy (XPS, Model: Escalab Xi + −, Make: Thermo Scientific, West Sussex, UK). The bonding characteristics and functional groups of the catalysts were identified through Fourier transform infrared spectroscopy (FTIR) using a Shimadzu IR Prestige-21 Fourier Transform Infrared 8300 spectrometer, Columbia, MD, USA equipped with a mercury–cadmium–telluride (MCT) detector. Finally, the thermal stability and heat flow in the catalysts were analyzed using thermogravimetric analysis and differential scanning calorimetry (TGA–DSC, Model: TA Instruments, New Castle, DE, USA).

### 3.6. FTS Process

For the FTS, ~0.16 g of the catalyst was loaded into the microreactor using the packing method, which optimizes the use of free volume within the microchannels. To secure and prevent its escape into the reaction line, both the inlet and outlet of the microreactor were packed with quartz wool and sealed with ¼-inch Swagelok VCR filter gaskets. The loaded microreactor was then carefully positioned within a custom-designed reactor block, as illustrated in Figure 12, and integrated into the reaction line using ¼-inch female Swagelok fittings [65,66].

**Figure 13:**
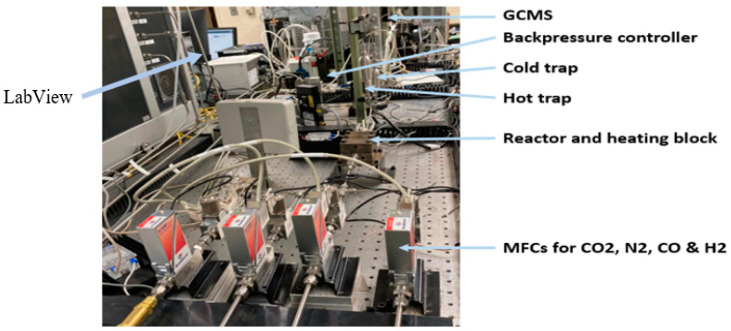
Experimental setup for FT synthesis.

The FTS experiments were executed using a LabVIEW-controlled setup that allowed rigid management of the reaction conditions, depicted in Figure 13. The syngas mixture (H_2_ and CO) and carrier gas (N_2_) flow were regulated by a pre-calibrated Bronkhorst (maximum calibration at 20 sccm) mass flow controllers. Pressure levels, both upstream and downstream, were monitored using Bronkhorst pressure gauges. The entire system was automated through LabVIEW 2018 software. Analytical assessments of the products formed were conducted using Agilent Technologies 7890B gas chromatography and 5977 MSD systems, Wilmington, DE, USA. Before initiating the experiments, the catalyst within the microreactor was reduced in situ at 350 °C overnight under a hydrogen atmosphere. The actual FTS reaction was conducted with a syngas ratio of H_2_/CO = 2 at a fixed gas hourly space velocity (GHSV) of 12,000 h^−1^, maintaining H_2_ and CO flow rates at 4 and 2 mL/min, respectively, and a N_2_ flow rate at 1.5 mL/min [61]. CO conversion and hydrocarbons selectivity were calculated using Equations (1)–(4):

(1) $$X_{CO}\%=\frac{F_{CO,in}-F_{CO,out}}{F_{CO,in}}\times 100$$

where *F_CO,in_* = inlet molar flow rate of CO and *F_CO,out_* = outlet molar flow rate of CO;

(2) $${CH}_{4}\;Selectivity\;\left(\%\right)=\frac{m{CH}_{4}}{m{CH}_{4}+2mC_{2}H_{6}+3m{C_{3}H}_{8}+4m{C_{4}H}_{10}}\times 100$$

(3) $${C_{2}H}_{6}\;Selectivity\;\left(\%\right)=\frac{2m{C_{2}H}_{6}}{m{CH}_{4}+2mC_{2}H_{6}+3m{C_{3}H}_{8}+4m{C_{4}H}_{10}}\times 100$$

(4) $${C_{3}H}_{8}\;Selectivity\;\left(\%\right)=\frac{3m{C_{3}H}_{8}}{m{CH}_{4}+2mC_{2}H_{6}+3m{C_{3}H}_{8}+4m{C_{4}H}_{10}}\times 100$$

where *m* = number of carbon atoms present in each product.

## 4. Stability Studies of All Catalysts

**Figure 14:**
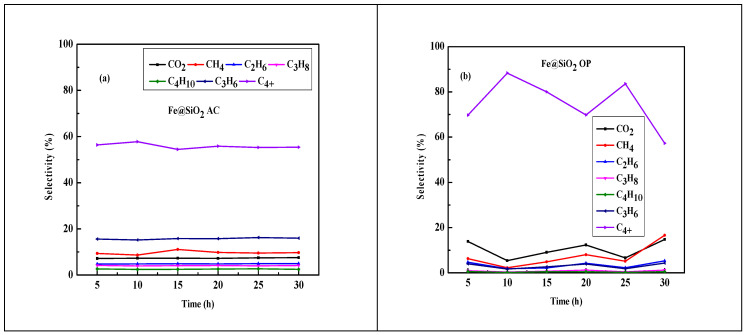
Time-on-stream behavior of Fe@SiO_2_ (AC); Fe@SiO_2_ (OP) catalysts (conditions: H_2_/CO = 2, 20 bar, GHSV = 12,000 h^−1^ at 320 °C for 30 h and N_2_ = 1.5 mL/min).

Long-term performance tests conducted over 30 h at 320 °C revealed relatively stable CO conversion of 60–65% for the Fe@SiO_2_ (AC) catalyst, whereas the Fe@SiO_2_ (OP) catalyst showed lower and less stable conversion of 6–16% (shown in Figure 14). This result is consistent with the findings from TPR, BET, and XRD analyses. It also suggests a higher degree of reduction of iron oxide contributes to greater FTS activity due to more Fe-active sites [67,68,69]. This can be attributed to the reduction degree (55.64%) of the Fe@SiO_2_ (AC) catalyst greatly influencing the CO conversion and stability of the catalyst.

The product selectivity of hydrocarbons for all catalysts is shown in Figure 14a,b. Figure 14 shows the higher propene selectivity (16%) compared to the rest of the products (less than 12%). The structure of the core–shell helps protect the iron metal particles from oxidation and inhibits the growth of iron particles during activation of the catalysts and reactions [70]. Figure 14b shows the product selectivity for the Fe@SiO_2_ (OP) catalyst. The C_4+_ selectivity is higher compared to the rest of the products. This indicates that this catalyst is favored for the WGS reaction.

Our FT results suggest that the structure of core–shell catalysts promote the products shifting towards the heavier hydrocarbons (C_4+_). In addition, the presence of SiO_2_ in the shell of the Fe-based core–shell catalysts do not overcome the surface basicity; however, the active site of Fe plays another role in hydrocracking, which includes hydrogenation, double-bond migration, and skeletal isomerization [71].

### 4.1. Spent Catalyst Characterization

#### 4.1.1. SEM Analysis of Spent Catalysts

**Figure 15:**
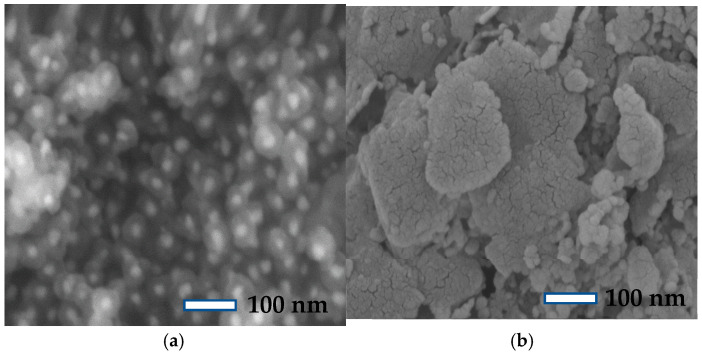
SEM images of spent catalysts: (**a**) Fe@SiO_2_ (AC); (**b**) Fe@SiO_2_ (OP).

Figure 15 shows the SEM images of all spent catalysts. The surface morphology of each catalyst changed after time-on-stream studies. More significantly, the morphologies of the Fe@SiO_2_ (AC) catalyst changed little after the stability studies (Figure 15a). The particle size is reduced; however, no agglomeration is observed for this catalyst. In contrast, agglomeration of particles is observed for the Fe@SiO_2_ (OP) catalyst (Figure 15b), and coke formation is visible over the catalyst surface after the FTS reactions. Coke formation is also evident from the decrease in catalyst activity.

#### 4.1.2. TGA-DSC Analysis of Spent Catalysts

In order to find coke deposition during Fischer–Tropsch reactions, TGA-DSC analysis was conducted in the presence of air up to 1000 °C for all spent catalysts [72]. The weight loss in these catalysts can be attributed to the buildup of carbides on the catalysts from the high-temperature Fischer–Tropsch reactions [52,72]. Weight gain can be caused by oxidation reactions. Oxidation reactions are generally exothermic in nature [73]. Since each catalyst was reduced prior to the Fischer–Tropsch reaction, we would expect to observe some weight gain during the spent catalyst analysis, although the weight loss due to coke burning would be greater than the weight gain from oxidation. Note that there may be silicon carbide contamination in these spent catalysts, although a sifter was used to filter out the silicon carbide.

**Figure 16:**
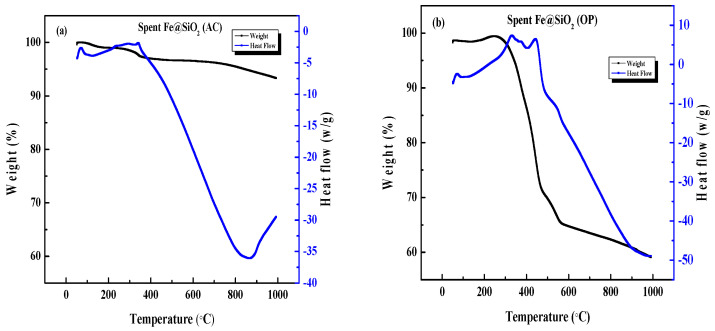
TGA-DSC thermograms of spent catalysts: (**a**) Fe@SiO_2_ (AC); (**b**) Fe@SiO_2_ (OP).

Figure 16a shows the TGA-DSC analysis of a spent Fe@SiO_2_ (AC) catalyst. There is an immediate upwards peak in the DSC curve representing an exothermic process, corresponding to slight weight gain that suggests oxidation [73]. This is followed by consistent weight loss for the spent catalyst, corresponding with a downward-sloping DSC curve with a minimum at 900 °C [52,72]. This can be attributed to the burning off of coke from the spent catalyst. The final weight of the spent catalyst is 92% of the original sample weight, suggesting that there was not excessive coking during the F-T reaction of this sample.

Figure 16b shows the TGA-DSC analysis of the spent Fe@SiO_2_ (OP) catalyst. This catalyst, as observed with the Fe@SiO_2_ (AC) catalyst, shows slight weight gain early in the analysis followed by a considerable weight loss, comparable to that of the spent Fe@SiO_2_ (AC) catalyst. In the DSC curve, an exothermic peak corresponds to weight gain, suggesting that the weight gain is from reoxidation of the sample. The weight loss corresponds to a minimum in the DSC curve, which suggests that the weight loss is from iron carbides that were produced during the reaction. This weight loss of the catalyst after removal of coke was ~7% of the original weight, showing less coking than that observed with the spent Fe@SiO_2_ (AC) catalyst—in other words, considerably more coking than that observed for the spent Fe@SiO_2_ (OP) catalyst.

#### 4.1.3. XRD Analysis of Spent Catalysts

**Figure 17:**
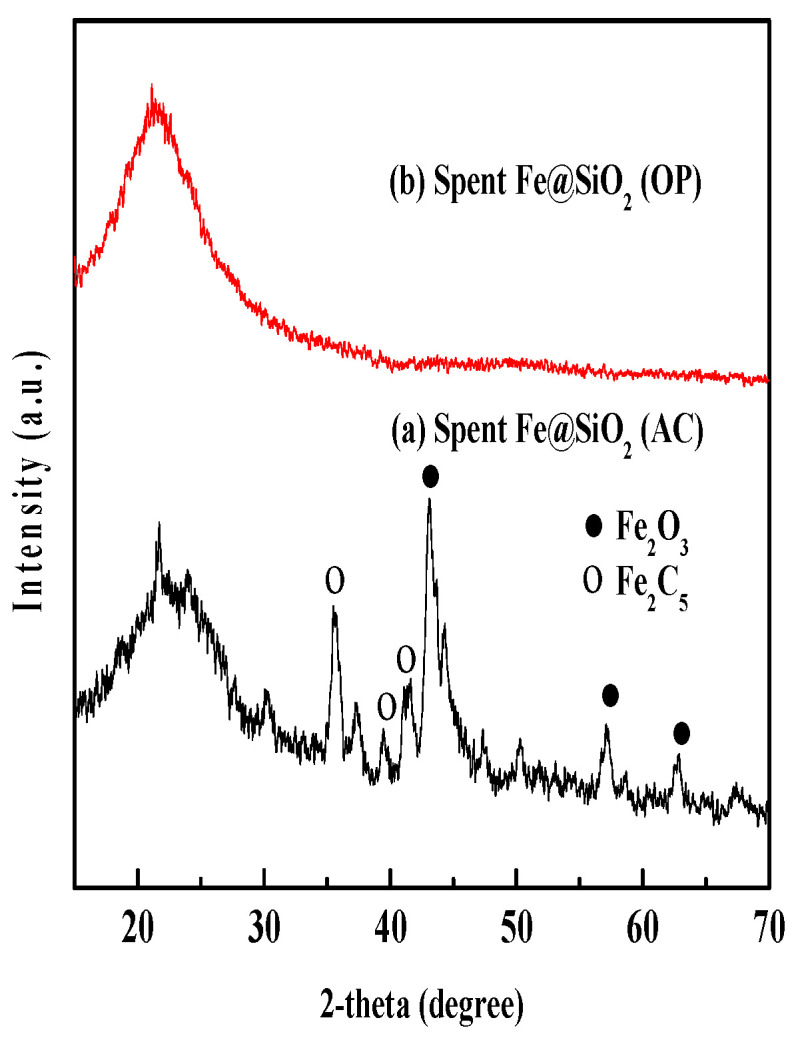
Wide-angle XRD patterns of spent catalysts: (a) Fe@SiO_2_ (AC); (b) Fe@SiO_2_ (OP).

Figure 17 shows wide-angle XRD analyses of all spent catalysts. The diffraction peaks of Fe_2_O_3_ with rhombohedral structure are observed at 43.25° (202), 57.09° (122), and 62.36° (214) for the spent Fe@SiO_2_ (AC) catalyst. The diffraction peaks matched those reported in the JCPDS-24-0072 database. The crystal size (8.98 nm) was reduced after the time-on-stream study. The crystal size was calculated using a modified Scherrer equation [36]. Some peaks of iron carbide (Fe_2_C_5_) with monoclinic structure are observed at 35.6° (002), 39.33° (020), and 40.9° (112). These peaks matched the JCPDS-36-1248 database. No crystals are observed for the spent Fe@SiO_2_ (OP) catalyst. This indicates that an amorphous structure is formed for the spent Fe@SiO_2_ (OP) catalyst. The intensity of each metal oxide peak is reduced.

### 4.2. Comparative Study of Activity of Different Fe-Based Catalysts

We have compared our catalyst activity in terms of CO conversion and product selectivity with other Fe-based catalysts. Table 5 shows the comparative results of different catalyst performances along with our catalyst. Clearly, the Fe@SiO_2_ (AC) catalyst shows higher performance and stability.

## 5. Conclusions

The FTS reactions performed in 3D-printed SSMR highlighted the critical role of catalyst synthesis methods and structural properties in influencing catalytic performance. These findings underscore the efficacy of the autoclave method over the one-pot method, particularly in terms of particle dispersion and catalytic activity, as corroborated by TPR, TEM, XRD, and SEM-EDX analyses. Autoclave synthesis resulted in the formation of a large-surface-area Fe@SiO_2_ catalyst with a mesoporous structure, as suggested by XRD and BET surface area studies. The XRD and TEM characterization studies suggested that the metal particles were well distributed in the Fe@SiO_2_ (AC) catalysts. When the two techniques are compared, the AC synthesis is better, because OP synthesis yields some agglomeration and collapsing of particles in the Fe@SiO_2_ (OP) catalyst. In addition, H_2_-TPR results clearly show that the synthesis method plays an important role in the reduction of metal oxide to produce the active sites for FTS. The TPR analysis is consistent with our TEM and XRD studies.

SEM-EDX and TEM results show a hexagonal matrix containing porous surface morphology with uniform metal ion distribution. 3D-printed SSMR was used to study the activity of core–shell catalysts in FTS at 20-bar pressure [65]. Although all catalysts displayed similar trends in CO conversion with respect to increased temperature, the maximum CO conversion was obtained as follows: Fe@SiO_2_ (AC) (85%) and Fe@SiO_2_ (OP) (22%). The selectivity towards olefins and C_4+_ hydrocarbons besides CH_4_ and CO_2_ was highest at temperatures below 300 °C and followed the order: Fe@SiO_2_ (OP) > Fe@SiO_2_ (AC). Based on these results, the Fe@SiO_2_ (AC) catalyst performed very well, considering the maximum CO conversion and favorable product selectivity. This is most likely due to the mesoporous hexagonal core–shell structure and large surface area of the AC catalyst. 3D-printed SSMR enables the addressing of crucial points of FTS and ease of catalyst screening and development in a microfluidic system.

The characterization of spent catalysts further demonstrates the resilience of Fe@SiO_2_ (AC) against coking. The SEM images and TGA-DSC analyses reflected small amounts of coke deposition on Fe@SiO_2_ (AC), while the Fe@SiO_2_ (OP) catalyst exhibited significant coke deposition, which led to agglomeration and lower activity in FTS.

These findings highlight the advantages of the autoclave method for synthesizing effective core–shell catalysts and demonstrate the importance of structural design to enhance catalyst activity and stability. The better stability and resistance to coking of the Fe@SiO_2_ (AC) catalyst make it a promising catalyst for long-term applications in FTS.

## Figures and Tables

**Table 1:** BET analysis of core–shell catalysts: Fe@SiO_2_ (OP); Fe@SiO_2_ (AC).

Catalyst	Surface Area (m ^2^/g)	Pore Volume (cc/g)	Pore Diameter (nm)
**Fe** **@SiO_2_ (OP)**	106	0.33	12.6
**Fe** **@SiO_2_ (AC)**	617	0.58	3.8

**Table 2:** Crystal size calculation * based on XRD data.

Catalyst	Avg. Fe_2_O_3_ Crystal Size (nm)	Avg. Fe_3_O_4_ Crystal Size (nm)
**Fe** **@SiO_2_ (OP)**	-	20.34
**Fe** **@SiO_2_ (AC)**	20.45	-

* Using modified Scherrer equation [36].

**Table 3:** SEM-EDS analysis of synthesized catalysts.

Catalyst	Metal Loading (wt. %) Fe	Silica (Si) Loading (wt. %)	Oxygen (O) Loading (wt. %)
**Fe** **@SiO_2_ (OP)**	28.92	19.1	51.98
**Fe** **@SiO_2_ (AC)**	26.87	27.79	45.34

**Table 4:** H_2_ consumption by different catalysts.

Catalyst	H_2_ Consumption (mmol/g) ^a^	Reduction Degree (%) ^b^
**Fe@SiO_2_ (AC)**	0.27	55.64
**Fe@SiO_2_ (OP)**	0.13	14.11

^a^ H_2_ consumption (mmol/g) was defined as the measured amount of H_2_ consumption in TPR peak/theoretical H_2_ consumption (mmol H_2_/g) × 100. ^b^ The degree of reduction (%) was defined according to the literature [49] as the measured amount of H_2_ consumption between 150–400 °C in TPR peak/theoretical H_2_ consumption (mmol H_2_/g) × 100.

**Table 5:** Activity of various Fe-based catalysts in FTS process.

Catalyst	Synthesis Method	CO Conversion (%)	Product Selectivity	Reference
FeK@SiO_2_-GC (Graphite carbon)	Graphitic carbon coating	80–90	High olefin (approx. 20%)	[28]
SAPO-34@Fe_3_C	Zeolite encapsulation	70	Light olefins (>18%)	[29]
Na-Mn-Fe/ZrO_2_	Solid state	42	High C_5+_ (60%)	[74]
CoFe/NC	Hydrothermal	40.9	High C_5+_ (66.1%)	[75]
Fe@NC/CS	Sol impregnation	56.2	High olefin (approx. 19.6%)	[76]
Fe@SiO_2_ (AC)	Autoclave	85	High olefin (16% propene), moderate C_4+_	This work

## Data Availability

The original contributions presented in this study are included in this article. Further inquiries can be directed to the corresponding author.

## Supplementary Materials

The following supporting information can be downloaded at https://www.mdpi.com/article/10.3390/molecules30020280/s1. Scheme S1: Preparation of Fe_3_O_4_ nanoparticles (core); Scheme S2: Preparation of SiO_2_ (shell); Figure S1: FTIR analyses of both catalysts: Fe_3_O_4_@SiO_2_ (OP); Fe_2_O_3_@SiO_2_ (AC); Figure S2: C1s of Fe_2_O_3_@SiO_2_ (AC); Figure S3: O1s of Fe_2_O_3_@SiO_2_ (AC); Figure S4: C1s of Fe_3_O_4_@SiO_2_ (OP); Figure S5: O1s of Fe_3_O_4_@SiO_2_ (OP).
