# Supplementary material for: Effect of Preparation Conditions of Fe@SiO2 Catalyst on Its Structure Using High-Pressure Activity Studies in a 3D-Printed SS Microreactor
Source: Molecules. 2025 Jan 13;30(2):280. doi: 10.3390/molecules30020280 (PMC11767676; doi:10.3390/molecules30020280)
Supplement: Supplementary file 1 [file molecules-30-00280-s001.zip › molecules-3374218-supplementary.pdf]

Effect of Preparation conditions of Fe@SiO_2 Catalyst on its Structure Using High-Pressure Activity Studies in a 3-D Printed SS Microreactor

Meric Arslan [1], Sujoy Bepari [2], Juvairia Shajahan [3], Saif Hassan[2], Debasish Kuila [1, 2, 3]

1 Department of Applied Science and Technology, North Carolina Agricultural and Technical State University, Greensboro, North Carolina, 27411, United States

2 Department of Chemistry, North Carolina Agricultural and Technical State University, Greensboro, North Carolina, 27411, United States

3 Joint School of Nanoscience and Nanoengineering, North Carolina Agricultural and Technical State University, Greensboro, NC 27411, USA

*Correspondence: dkuila@ncat.edu

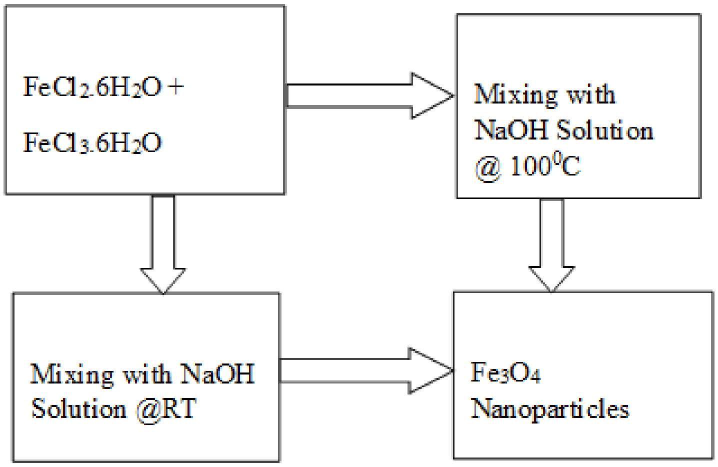

Scheme S1 Preparation of Fe_3O_4 nanoparticles (Core)

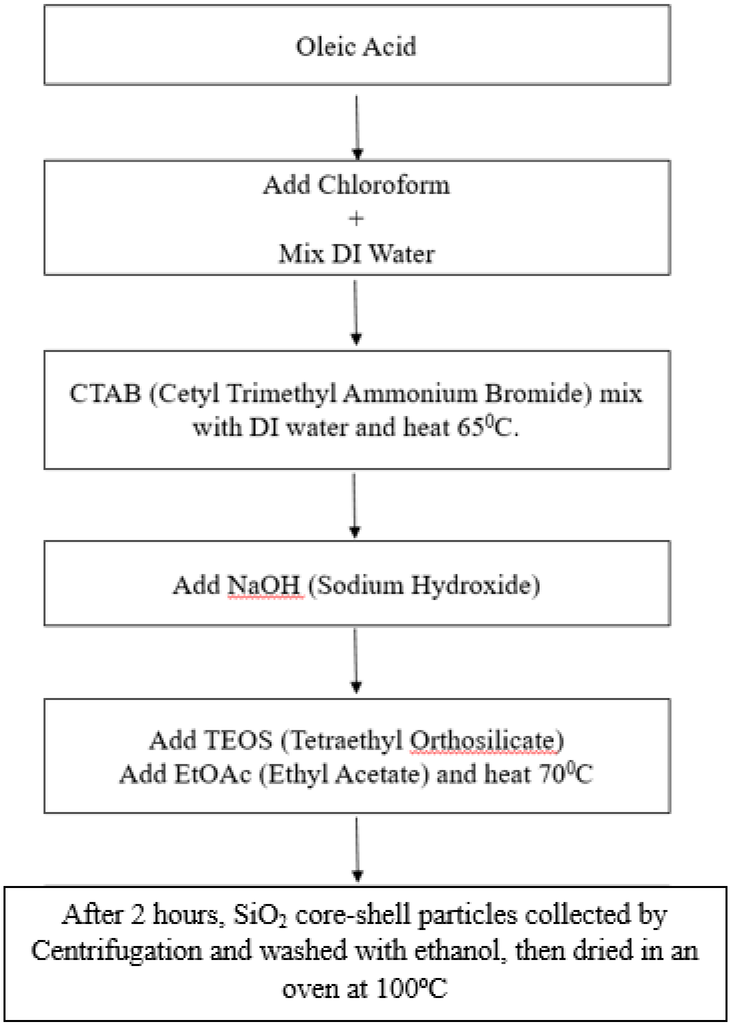

Scheme S2 Preparation of SiO_2 (Shell)

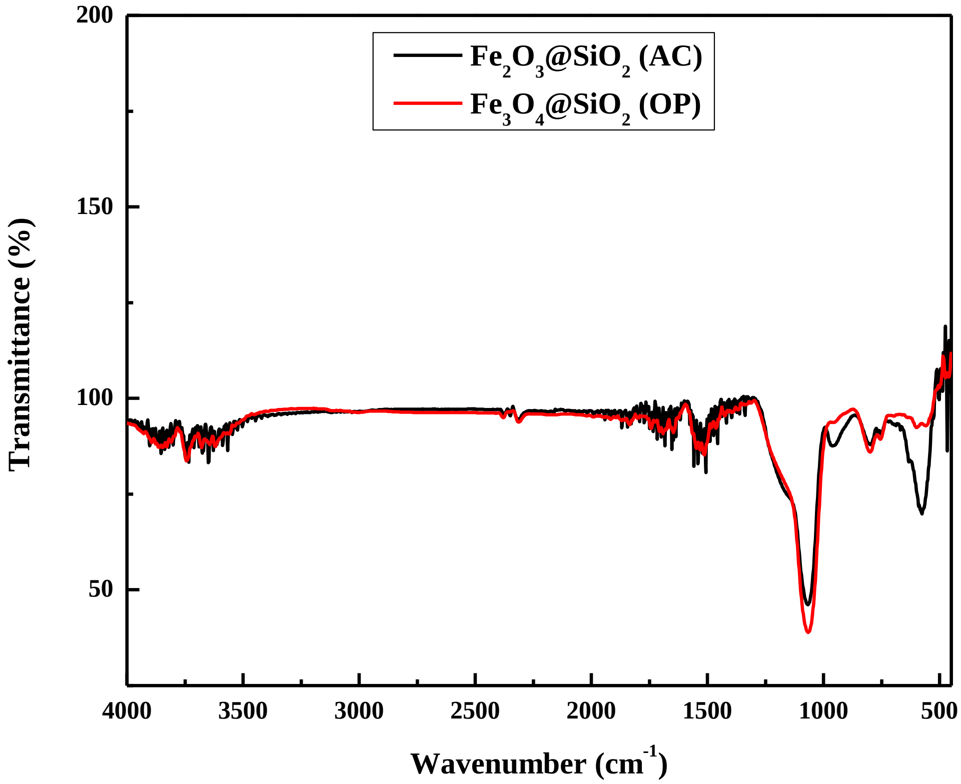

Figure S1 FTIR analyses of all catalysts $Fe_3O_4@SiO_2$ (OP); $Fe_2O_3@SiO_2$ (AC)

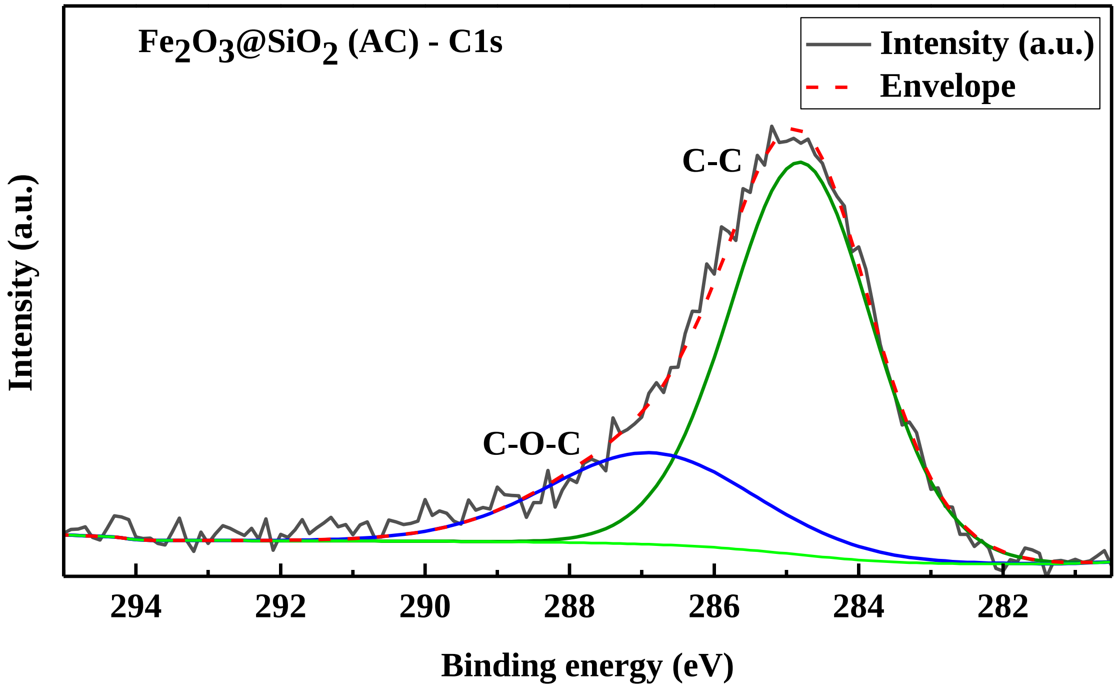

Figure S2 C1s of $Fe_2O_3@SiO_2$ (AC)

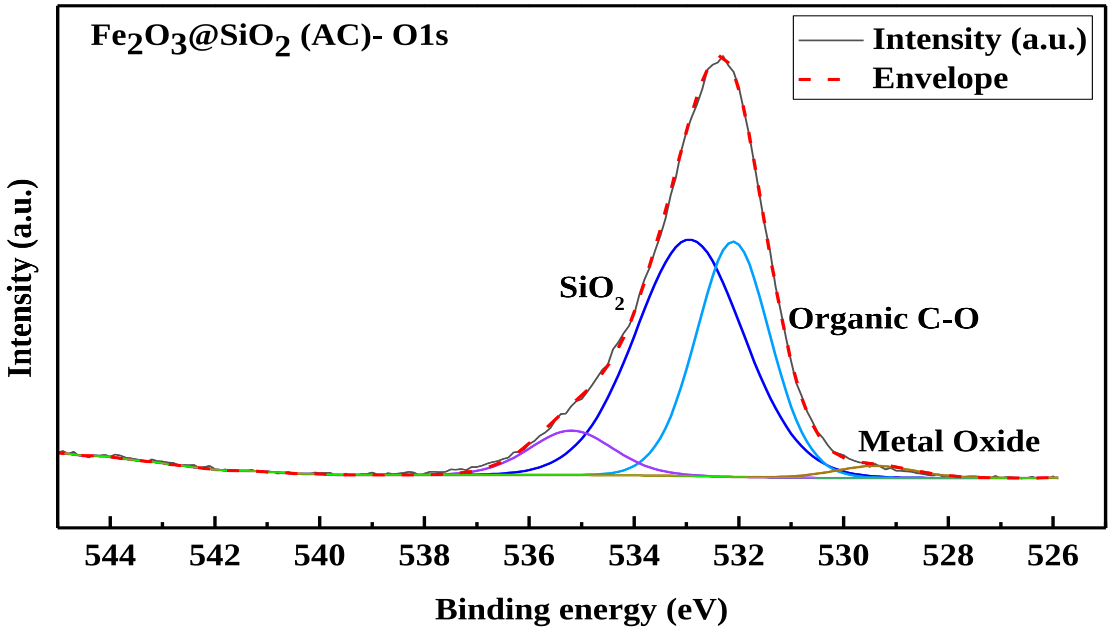

Figure S3 O1s of $Fe_2O_3@SiO_2$ (AC)

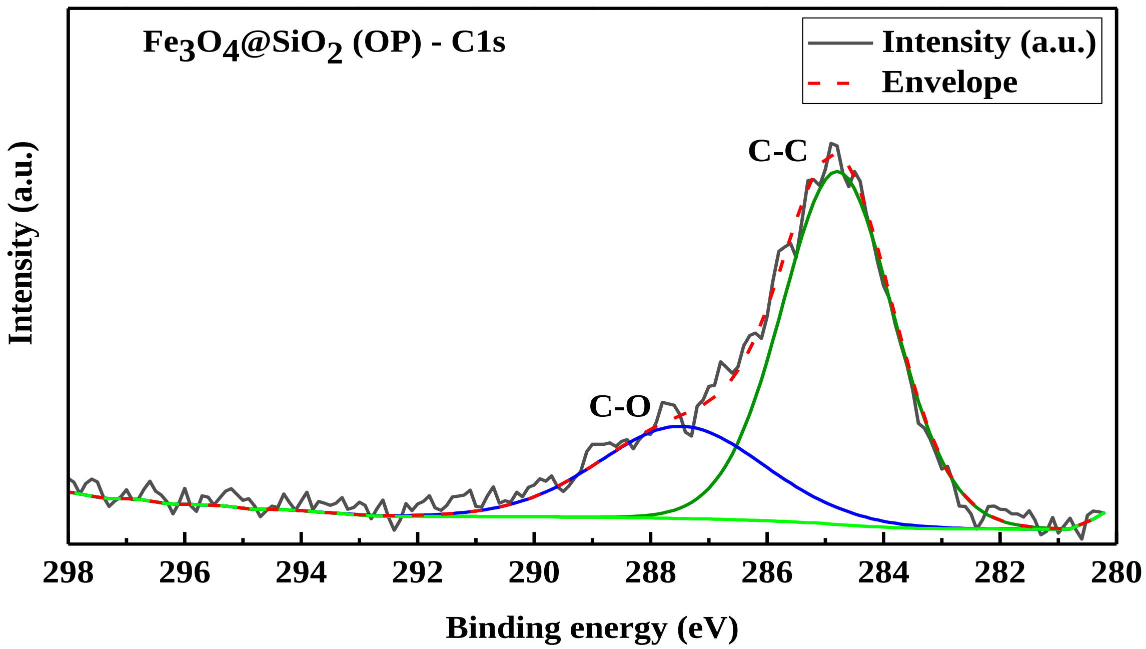

Figure S4 C1s of $Fe_3O_4@SiO_2$ (OP)

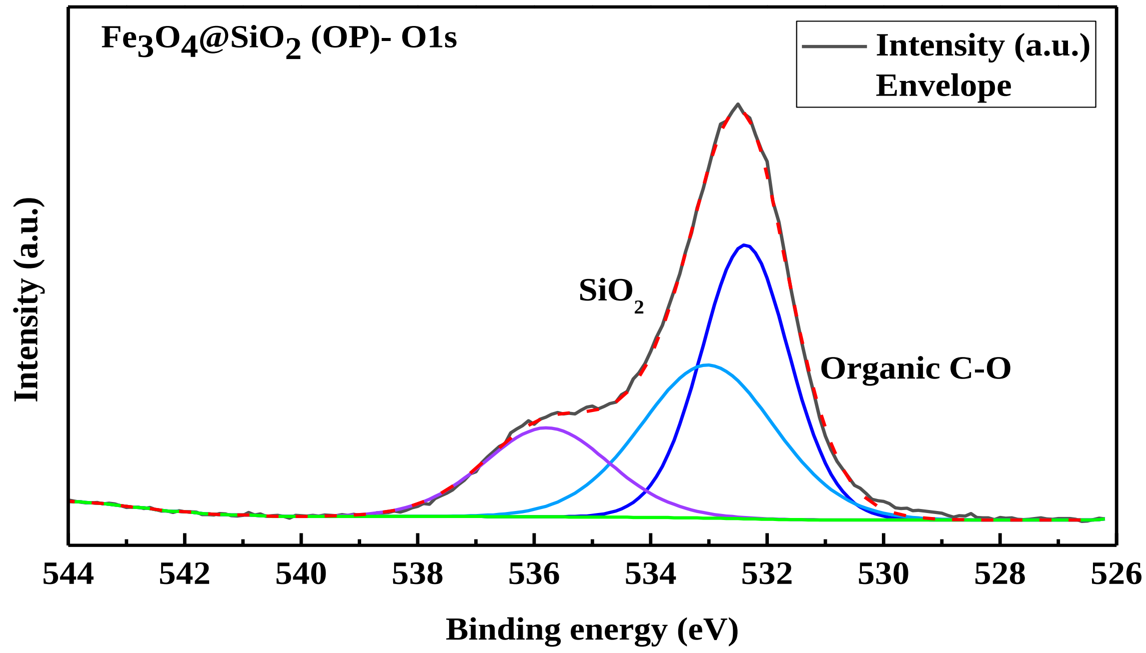

Figure S5 O1s of $Fe_3O_4@SiO_2$ (OP)
